# Supplementary material for: Adult patients with autoinflammation of unknown origin partially phenocopy the immune presentation of Still’s disease
Source: Nat Commun. 2026 Apr 1;17:4670. doi: 10.1038/s41467-026-70895-1 (PMC13201634; doi:10.1038/s41467-026-70895-1)
Supplement: Supplementary file 1 — Supplementary Information [file 41467_2026_70895_MOESM1_ESM.pdf]

Supplementary information for:

Adult patients with autoinflammation of unknown origin partially phenocopy the immune presentation of Still's disease.

Rafael Veiga[†], Leana De Vuyst[†], Christophe Poulet[†], Julika Neumann, Leoni Bücken, Teresa Prezzemolo, Mathijs Willemsen, Steven Vanderschueren, Patrick Matthys, Emna Chabaane, Maximilien Fléron, Gaël Cobraiville, Dominique Baiwir, Gabriel Mazzucchelli, Immunome Project Consortium for Autoinflammatory Disorders (ImmunAID), Bruno Fautrel, Carine Wouters[*], Dominique De Seny[*], Stephanie Humblet-Baron[*], Adrian Liston[*]

^ Supplementary Note 1:

ImmunAID consortium members : Vassili Soumelis, Arturo Hernandez Cervantes, Thomas Henry, Irina Giurgea, Serge Amselem, Sonia Karabina, Gilles Hayem, Alexandre Belot, Yvan Jamilloux, Eric Hachulla, Thierry Martin, Marie-Elise Truchetet, Nicolas Poursac, Antonine Néel (INSERM), Bruno Fautrel, Stéphane Mitrovic, Emna Chabaane, David Saadoun, Matheus Vieira, Isabelle Koné-Paut, Nasima Matsa, Perrine Dusser, Jean-David Bouaziz, Thibault Mahevas, Sophie Georgin-Lavialle, Keshia Koutekissa, Léa Savey, Pierre Quartier (APHP), Savvas Savvides, Stephanie Humblet-Baron, Adrian Liston, Jeroen Raes (VIB), Carine Wouters, Steven Vanderschueren, Mieke Gouwy, Paul Proost, Patrick Matthys (KU Leuven), Søren Brunak, Isabella Friis Jørgensen, Andres Jimenez Kaufmann, Sara Garcia (UCPH), Philippe Hupé, Fanny Coffin, Apolline Gallois, Henri de Soyres, Julien Roméjon (Institut Curie), Aura Moreno Vega (Owkin), Marc Dubourdeau (Ambiotis), Karoline Krause, Dirk Foell, Christoph Kessel (WWU), Katerina Laskari, Andreakos Evangelos (BRFAA), Paul Van Daele, Clementien Vermont, Rogier van Wijck, Sigrid Swagemakers, Peter van der Spek, Stefan Erkeland, Harmen van de Werken, Yvonne Mueller, Peter Katsikis (Erasmus MC), Michael Hofer, Cem Gabay (Unige), Sinisa Savic, Michael F. McDermott (University Leeds), Helen Lachmann (UCL), Costas Papaloukas, Dimitrios I. Fotiadis (UOI), Dominique de Seny, Christophe Poulet (ULiege), Haner Direskeneli, Seza Ozen, Umut Kalyoncu (Hacettepe), Tadej Avcin (UMCL), Jordi Anton, Violeta Bittermann, Alina Boteanu (HSJD), Marco Francesco Natale, Antonella Insalaco, Emanuele Bizzi, Sonia Caccia, Luca Cantarini, Paolo Sfriso (OPBG), Jean-François Deleuze, Anne Boland (CEA), Sarhan Yaiche (ECRIN).

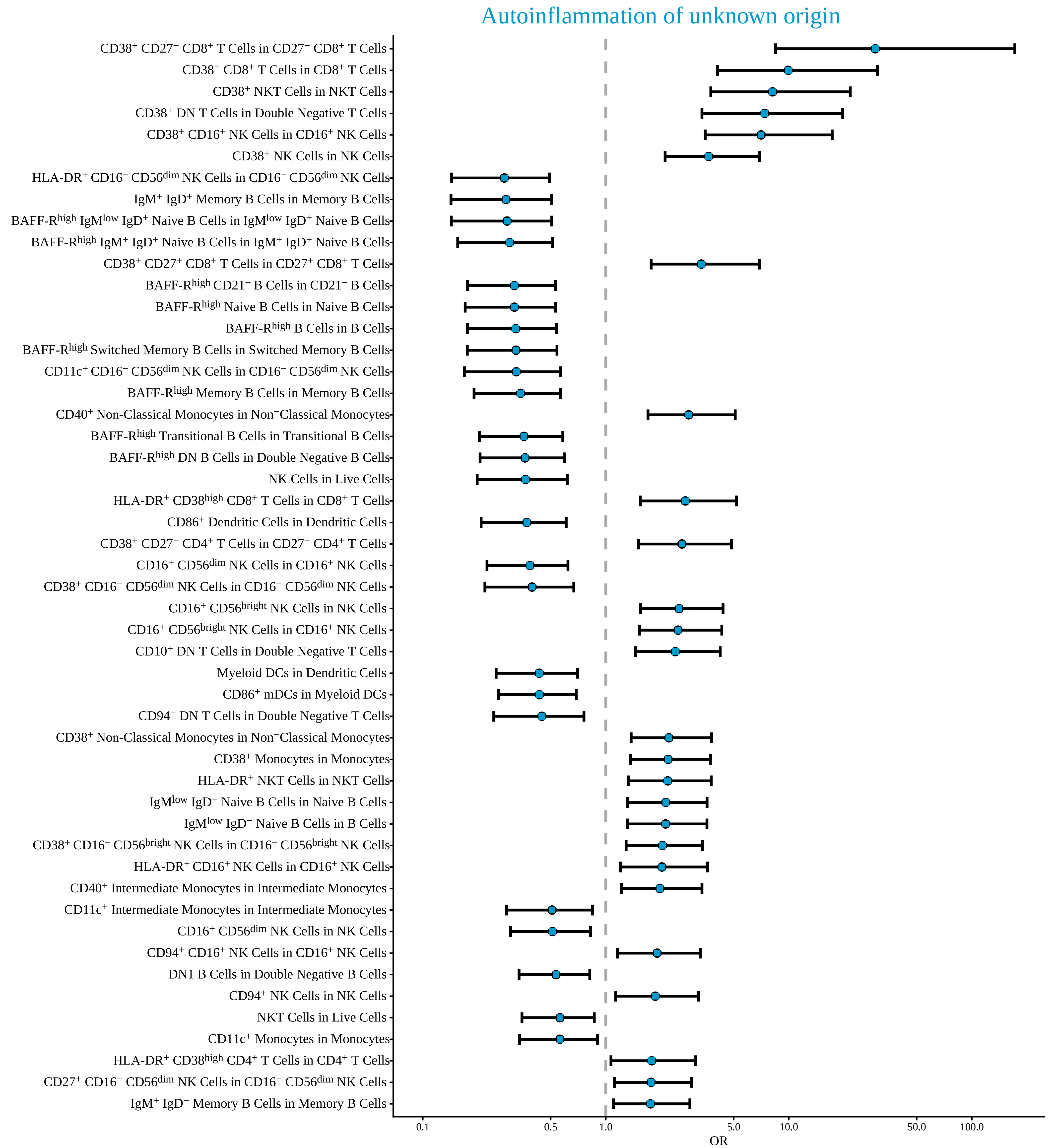
Autoinflammation of unknown origin
CD38+ CD27− CD8+ T Cells in CD27− CD8+ T Cells
CD38+ CD8+ T Cells in CD8+ T Cells
CD38+ NKT Cells in NKT Cells
CD38+ DN T Cells in Double Negative T Cells
CD38+ CD16+ NK Cells in CD16+ NK Cells
CD38+ NK Cells in NK Cells
HLA-DR+ CD16− CD56dim NK Cells in CD16− CD56dim NK Cells
IgM+ IgD+ Memory B Cells in Memory B Cells
BAFF-Rhigh IgMlow IgD+ Naive B Cells in IgMlow IgD+ Naive B Cells
BAFF-Rhigh IgM+ IgD+ Naive B Cells in IgM+ IgD+ Naive B Cells
CD38+ CD27+ CD8+ T Cells in CD27+ CD8+ T Cells
BAFF-Rhigh CD21− B Cells in CD21− B Cells
BAFF-Rhigh Naive B Cells in Naive B Cells
BAFF-Rhigh B Cells in B Cells
BAFF-Rhigh Switched Memory B Cells in Switched Memory B Cells
CD11c+ CD16− CD56dim NK Cells in CD16− CD56dim NK Cells
BAFF-Rhigh Memory B Cells in Memory B Cells
CD40+ Non-Classical Monocytes in Non−Classical Monocytes
BAFF-Rhigh Transitional B Cells in Transitional B Cells
BAFF-Rhigh DN B Cells in Double Negative B Cells
NK Cells in Live Cells
HLA-DR+ CD38high CD8+ T Cells in CD8+ T Cells
CD86+ Dendritic Cells in Dendritic Cells
CD38+ CD27− CD4+ T Cells in CD27− CD4+ T Cells
CD16+ CD56dim NK Cells in CD16+ NK Cells
CD38+ CD16− CD56dim NK Cells in CD16− CD56dim NK Cells
CD16+ CD56bright NK Cells in NK Cells
CD16+ CD56bright NK Cells in CD16+ NK Cells
CD10+ DN T Cells in Double Negative T Cells
Myeloid DCs in Dendritic Cells
CD86+ mDCs in Myeloid DCs
CD94+ DN T Cells in Double Negative T Cells
CD38+ Non-Classical Monocytes in Non−Classical Monocytes
CD38+ Monocytes in Monocytes
HLA-DR+ NKT Cells in NKT Cells
IgMlow IgD− Naive B Cells in Naive B Cells
IgMlow IgD− Naive B Cells in B Cells
CD38+ CD16− CD56bright NK Cells in CD16− CD56bright NK Cells
HLA-DR+ CD16+ NK Cells in CD16+ NK Cells
CD40+ Intermediate Monocytes in Intermediate Monocytes
CD11c+ Intermediate Monocytes in Intermediate Monocytes
CD16+ CD56dim NK Cells in NK Cells
CD94+ CD16+ NK Cells in CD16+ NK Cells
DN1 B Cells in Double Negative B Cells
CD94+ NK Cells in NK Cells
NKT Cells in Live Cells
CD11c+ Monocytes in Monocytes
HLA-DR+ CD38high CD4+ T Cells in CD4+ T Cells
CD27+ CD16− CD56dim NK Cells in CD16− CD56dim NK Cells
IgM+ IgD− Memory B Cells in Memory B Cells
0.1
0.5
1.0
5.0
10.0
50.0
100.0
OR

Supplementary Figure 1. Cells populations with high association with autoinflammation of unknown. Odds ratio and 95% confidence interval (point and error bars) of 50 highly-associated cell population frequency changes in patients with inflammation of unknown origin in relation to healthy individuals. Estimated by multivariable logistic regression adjusted by sex and age.

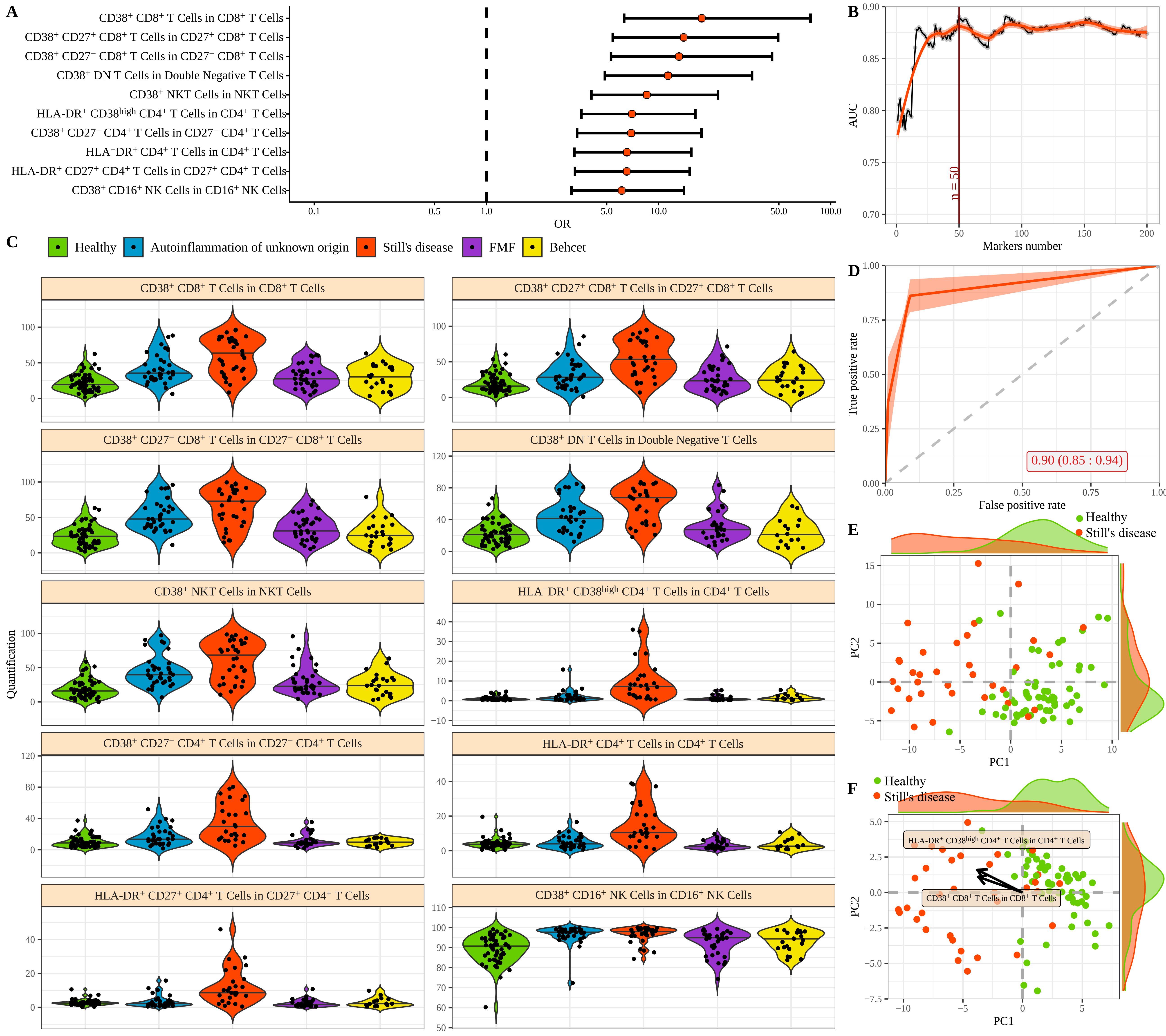

A
CD38+ CD8+ T Cells in CD8+ T Cells
CD38+ CD27+ CD8+ T Cells in CD27+ CD8+ T Cells
CD38+ CD27− CD8+ T Cells in CD27− CD8+ T Cells
CD38+ DN T Cells in Double Negative T Cells
CD38+ NKT Cells in NKT Cells
HLA-DR+ CD38high CD4+ T Cells in CD4+ T Cells
CD38+ CD27− CD4+ T Cells in CD27− CD4+ T Cells
HLA−DR+ CD4+ T Cells in CD4+ T Cells
HLA-DR+ CD27+ CD4+ T Cells in CD27+ CD4+ T Cells
CD38+ CD16+ NK Cells in CD16+ NK Cells
OR
B
AUC
n = 50
Markers number
C
Healthy
Autoinflammation of unknown origin
Still's disease
FMF
Behcet
Quantification
D
True positive rate
False positive rate
0.90 (0.85 : 0.94)
E
Healthy
Still's disease
PC1
PC2
F
Healthy
Still's disease
HLA-DR+ CD38high CD4+ T Cells in CD4+ T Cells
CD38+ CD8+ T Cells in CD8+ T Cells
PC1
PC2

Supplementary Figure 2. Immunological parameters associated with Still's disease. Patients with Still's disease (n=34) were compared against healthy individuals (n=58) by multivariate logistic regression of immunological parameters. Data from patients with autoinflammation of unknown origin (n=36), FMF (n=35) and Behçet (n=23) are shown as reference data only. **A)** Odds ratio and 95% confidence interval (point and error bars) of highly-associated cell population frequency changes in patients with Still's disease in relation to healthy individuals. Estimated by multivariable logistic regression adjusted by sex and age. **B)** Average and 95% confidence interval (red line and red band) of 200 times 10 fold cross-validation to evaluate a sufficient number of best cell populations, based on ability to adequately discern between Still's disease and healthy individuals. **C)** Frequency for highly-associated cell populations for Still's disease in relation to healthy individuals. Each dot represents a patient and each colour represents a condition. Bar indicates median, violin plot indicates data density. **D)** Average ROC curve with 95% confidence interval (red line and red band) of 10 fold cross-validation for Still's disease in relation to healthy individuals. ROC calculated using multi-variable logistic regression, adjusted by sex and age, considering the 82 cell populations with highest explanatory contribution. Area under ROC curve and confidence interval indicated on graph. **E)** First two PCA components of all cell populations in the dataset. Each dot represents an individual and each colour represents a condition. Histograms show distribution of values in Still's disease and healthy individuals. **F)** First two PCA components of 82 cell populations most highly associated for divergence between Still's disease and healthy individuals. Histograms show distribution of values in Still's disease and healthy individuals. The two arrows show the direction of distinct highly associated cell populations.

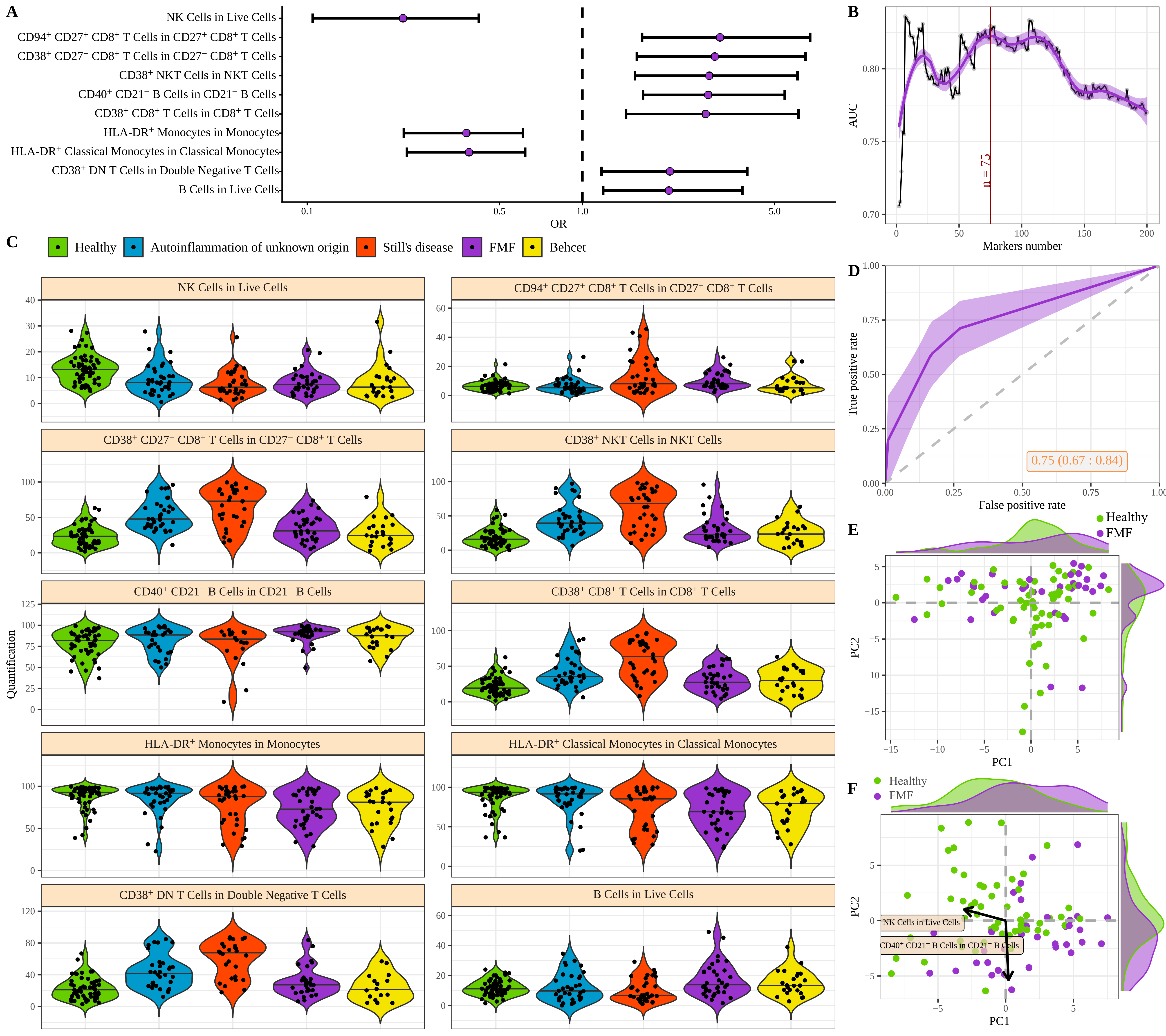

A
NK Cells in Live Cells
CD94+ CD27+ CD8+ T Cells in CD27+ CD8+ T Cells
CD38+ CD27− CD8+ T Cells in CD27− CD8+ T Cells
CD38+ NKT Cells in NKT Cells
CD40+ CD21− B Cells in CD21− B Cells
CD38+ CD8+ T Cells in CD8+ T Cells
HLA-DR+ Monocytes in Monocytes
HLA-DR+ Classical Monocytes in Classical Monocytes
CD38+ DN T Cells in Double Negative T Cells
B Cells in Live Cells
OR
B
AUC
n = 75
Markers number
C
Healthy
Autoinflammation of unknown origin
Still's disease
FMF
Behcet
Quantification
D
True positive rate
False positive rate
0.75 (0.67 : 0.84)
E
Healthy
FMF
PC1
PC2
F
Healthy
FMF
NK Cells in Live Cells
CD40+ CD21− B Cells in CD21− B Cells
PC1
PC2

Supplementary Figure 3. Immunological parameters associated with FMF. Patients with FMF (n=35) were compared against healthy individuals (n=58) by multivariate logistic regression of immunological parameters. Data from patients with autoinflammation of unknown origin (n=36), Still's disease (n=34) and Behçet (n=23) are shown as reference data only. **A)** Odds ratio and 95% confidence interval (point and error bars) of highly-associated cell population frequency changes in patients with FMF in relation to healthy individuals. Estimated by multivariable logistic regression adjusted by sex and age. **B)** Average and 95% confidence interval (purple line and purple band) of 200 times 10 fold cross-validation to evaluate a sufficient number of best cell populations, based on ability to adequately discern between FMF and healthy individuals. **C)** Frequency for highly-associated cell populations for FMF in relation to healthy individuals. Each dot represents a patient and each colour represents a condition. Bar indicates median, violin plot indicates data density. **D)** Average ROC curve with 95% confidence interval (purple line and purple band)l of 10 fold cross-validation for FMF in relation to healthy individuals. ROC calculated using multi-variable logistic regression, adjusted by sex and age, considering the 55 cell populations with highest explanatory contribution. Area under ROC curve and confidence interval indicated on graph. **E)** First two PCA components of all cell populations in the dataset. Each dot represents an individual and each colour represents a condition. Histograms show distribution of values in FMF and healthy individuals. **F)** First two PCA components of **55** cell populations most highly associated for divergence between FMF and healthy individuals. Histograms show distribution of values in FMF and healthy individuals. The two arrows show the direction of distinct highly associated cell populations.

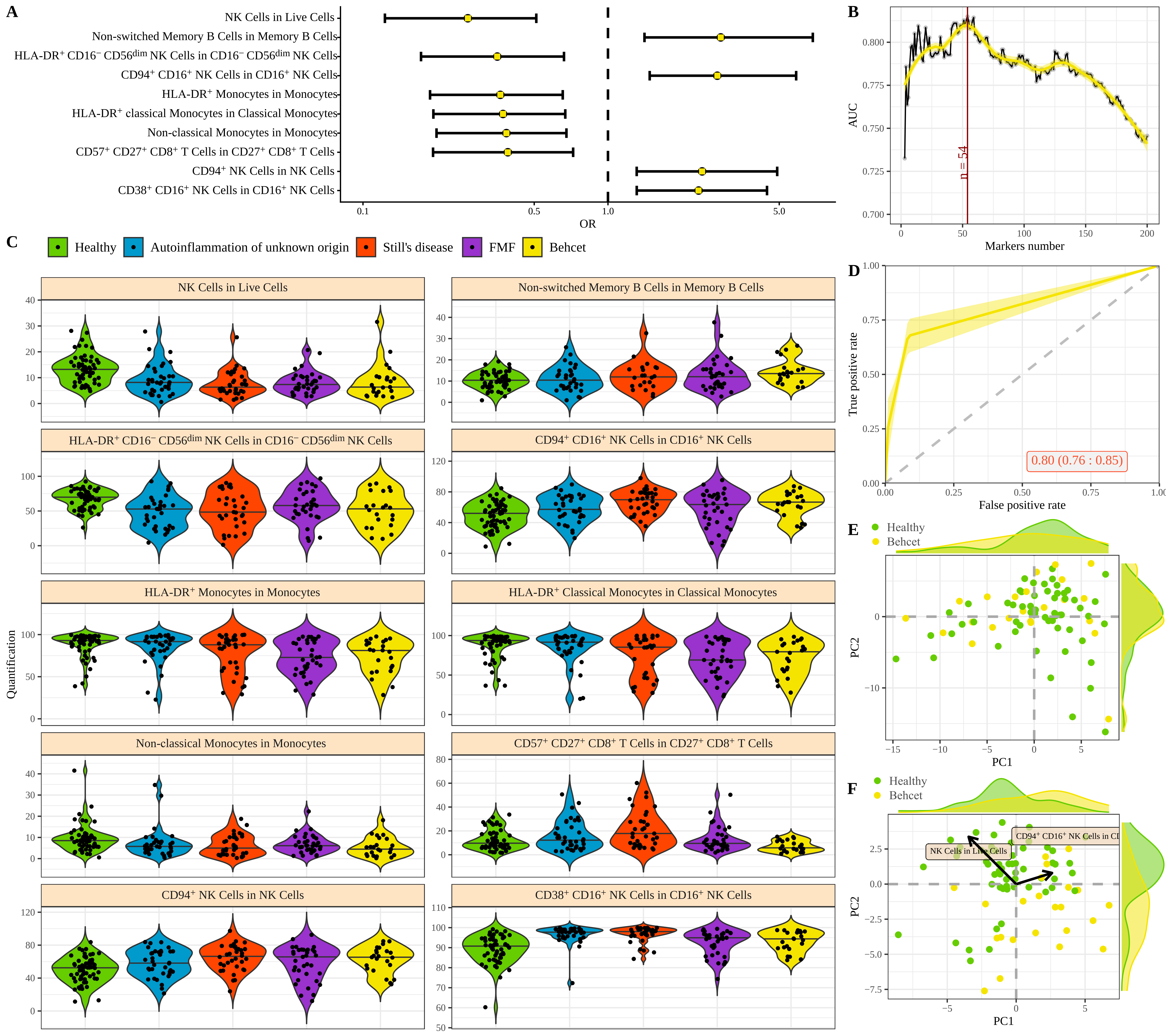

A
NK Cells in Live Cells
Non-switched Memory B Cells in Memory B Cells
HLA-DR+ CD16− CD56dim NK Cells in CD16− CD56dim NK Cells
CD94+ CD16+ NK Cells in CD16+ NK Cells
HLA-DR+ Monocytes in Monocytes
HLA-DR+ classical Monocytes in Classical Monocytes
Non-classical Monocytes in Monocytes
CD57+ CD27+ CD8+ T Cells in CD27+ CD8+ T Cells
CD94+ NK Cells in NK Cells
CD38+ CD16+ NK Cells in CD16+ NK Cells
OR
B
AUC
n = 54
Markers number
C
Healthy
Autoinflammation of unknown origin
Still's disease
FMF
Behcet
Quantification
HLA-DR+ Classical Monocytes in Classical Monocytes
D
True positive rate
False positive rate
0.80 (0.76 : 0.85)
E
Healthy
Behcet
PC1
PC2
F
NK Cells in Live Cells
CD94+ CD16+ NK Cells in CD

Supplementary Figure 4. Immunological parameters associated with Behçet's disease. Patients with Behçet's disease (n=23) were compared against healthy individuals (n=58) by multivariate logistic regression of immunological parameters. Data from patients with autoinflammation of unknown origin (n=36), Still's disease (n=34) and FMF (n=35) are shown as reference data only. **A)** Odds ratio and 95% confidence interval (point and error bars) of highly-associated cell population frequency changes in patients with Behçet in relation to healthy individuals. Estimated by multivariable logistic regression adjusted by sex and age. **B)** Average and 95% confidence interval (yellow line and yellow band) of 200 times 10 fold cross-validation to evaluate a sufficient number of best cell populations, based on ability to adequately discern between Behçet and healthy individuals. **C)** Frequency for highly-associated cell populations for Behçet in relation to healthy individuals. Each dot represents a patient and each colour represents a condition. Bar indicates median, violin plot indicates data density. **D)** Average ROC curve with 95% confidence interval (yellow line and yellow band) of 10 fold cross-validation for Behçet in relation to healthy individuals. ROC calculated using multi-variable logistic regression, adjusted by sex and age, considering the 61 cell populations with highest explanatory contribution. Area under ROC curve and confidence interval indicated on graph. **E)** First two PCA components of all cell populations in the dataset. Each dot represents an individual and each colour represents a condition. Histograms show distribution of values in Behçet and healthy individuals. **F)** First two PCA components of 61 cell populations most highly associated for divergence between Behçet and healthy individuals. Histograms show distribution of values in Behçet and healthy individuals. The two arrows show the direction of distinct highly associated cell populations.

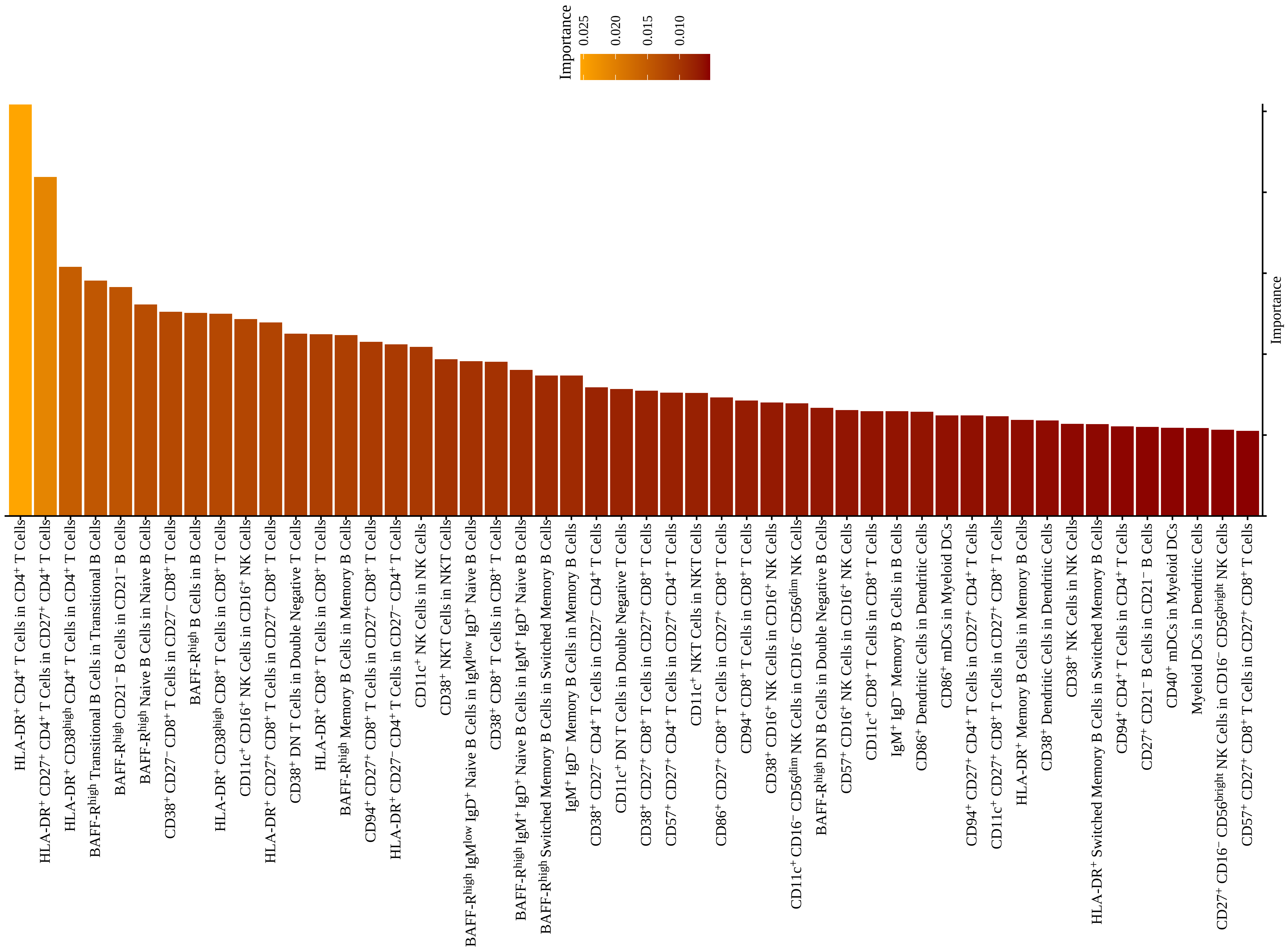
Importance
0.025
0.020
0.015
0.010
Importance
HLA-DR$^+$ CD4$^+$ T Cells in CD4$^+$ T Cells
HLA-DR$^+$ CD27$^+$ CD4$^+$ T Cells in CD27$^+$ CD4$^+$ T Cells
HLA-DR$^+$ CD38^{high} CD4$^+$ T Cells in CD4$^+$ T Cells
BAFF-R^{high} Transitional B Cells in Transitional B Cells
BAFF-R^{high} CD21$^-$ B Cells in CD21$^-$ B Cells
BAFF-R^{high} Naive B Cells in Naive B Cells
CD38$^+$ CD27$^-$ CD8$^+$ T Cells in CD27$^-$ CD8$^+$ T Cells
BAFF-R^{high} B Cells in B Cells
HLA-DR$^+$ CD38^{high} CD8$^+$ T Cells in CD8$^+$ T Cells
CD11c$^+$ CD16$^+$ NK Cells in CD16$^+$ NK Cells
HLA-DR$^+$ CD27$^+$ CD8$^+$ T Cells in CD27$^+$ CD8$^+$ T Cells
CD38$^+$ DN T Cells in Double Negative T Cells
HLA-DR$^+$ CD8$^+$ T Cells in CD8$^+$ T Cells
BAFF-R^{high} Memory B Cells in Memory B Cells
CD94$^+$ CD27$^+$ CD8$^+$ T Cells in CD27$^+$ CD8$^+$ T Cells
HLA-DR$^+$ CD27$^-$ CD4$^+$ T Cells in CD27$^-$ CD4$^+$ T Cells
CD11c$^+$ NK Cells in NK Cells
CD38$^+$ NKT Cells in NKT Cells
BAFF-R^{high} IgM^{low} IgD$^+$ Naive B Cells in IgM^{low} IgD$^+$ Naive B Cells
CD38$^+$ CD8$^+$ T Cells in CD8$^+$ T Cells
BAFF-R^{high} IgM$^+$ IgD$^+$ Naive B Cells in IgM$^+$ IgD$^+$ Naive B Cells
BAFF-R^{high} Switched Memory B Cells in Switched Memory B Cells
IgM$^+$ IgD$^-$ Memory B Cells in Memory B Cells
CD38$^+$ CD27$^-$ CD4$^+$ T Cells in CD27$^-$ CD4$^+$ T Cells
CD11c$^+$ DN T Cells in Double Negative T Cells
CD38$^+$ CD27$^+$ CD8$^+$ T Cells in CD27$^+$ CD8$^+$ T Cells
CD57$^+$ CD27$^+$ CD4$^+$ T Cells in CD27$^+$ CD4$^+$ T Cells
CD11c$^+$ NKT Cells in NKT Cells
CD86$^+$ CD27$^+$ CD8$^+$ T Cells in CD27$^+$ CD8$^+$ T Cells
CD94$^+$ CD8$^+$ T Cells in CD8$^+$ T Cells
CD38$^+$ CD16$^+$ NK Cells in CD16$^+$ NK Cells
CD11c$^+$ CD16$^-$ CD56^{dim} NK Cells in CD16$^-$ CD56^{dim} NK Cells
BAFF-R^{high} DN B Cells in Double Negative B Cells
CD57$^+$ CD16$^+$ NK Cells in CD16$^+$ NK Cells
CD11c$^+$ CD8$^+$ T Cells in CD8$^+$ T Cells
IgM$^+$ IgD$^-$ Memory B Cells in B Cells
CD86$^+$ Dendritic Cells in Dendritic Cells
CD86$^+$ mDCs in Myeloid DCs
CD94$^+$ CD27$^+$ CD4$^+$ T Cells in CD27$^+$ CD4$^+$ T Cells
CD11c$^+$ CD27$^+$ CD8$^+$ T Cells in CD27$^+$ CD8$^+$ T Cells
HLA-DR$^+$ Memory B Cells in Memory B Cells
CD38$^+$ Dendritic Cells in Dendritic Cells
CD38$^+$ NK Cells in NK Cells
HLA-DR$^+$ Switched Memory B Cells in Switched Memory B Cells
CD94$^+$ CD4$^+$ T Cells in CD4$^+$ T Cells
CD27$^+$ CD21$^-$ B Cells in CD21$^-$ B Cells
CD40$^+$ mDCs in Myeloid DCs
Myeloid DCs in Dendritic Cells
CD27$^+$ CD16$^-$ CD56^{bright} NK Cells in CD16$^-$ CD56^{bright} NK Cells
CD57$^+$ CD27$^+$ CD8$^+$ T Cells in CD27$^+$ CD8$^+$ T Cells

Supplementary Figure 5. Key immunological features driving machine learning-led disease identification. A multi-disease comparison was performed using a Random Forest algorithm to identify immune characteristics with discriminating potential between patients with autoinflammation of unknown origin (n=36), Still's disease (n=34), FMF (n=35) and Behçet's (n=23). Healthy individuals were not used in the model generation, and are shown as reference data only. Model importance for the 50 highest associated cell populations for disease discrimination.

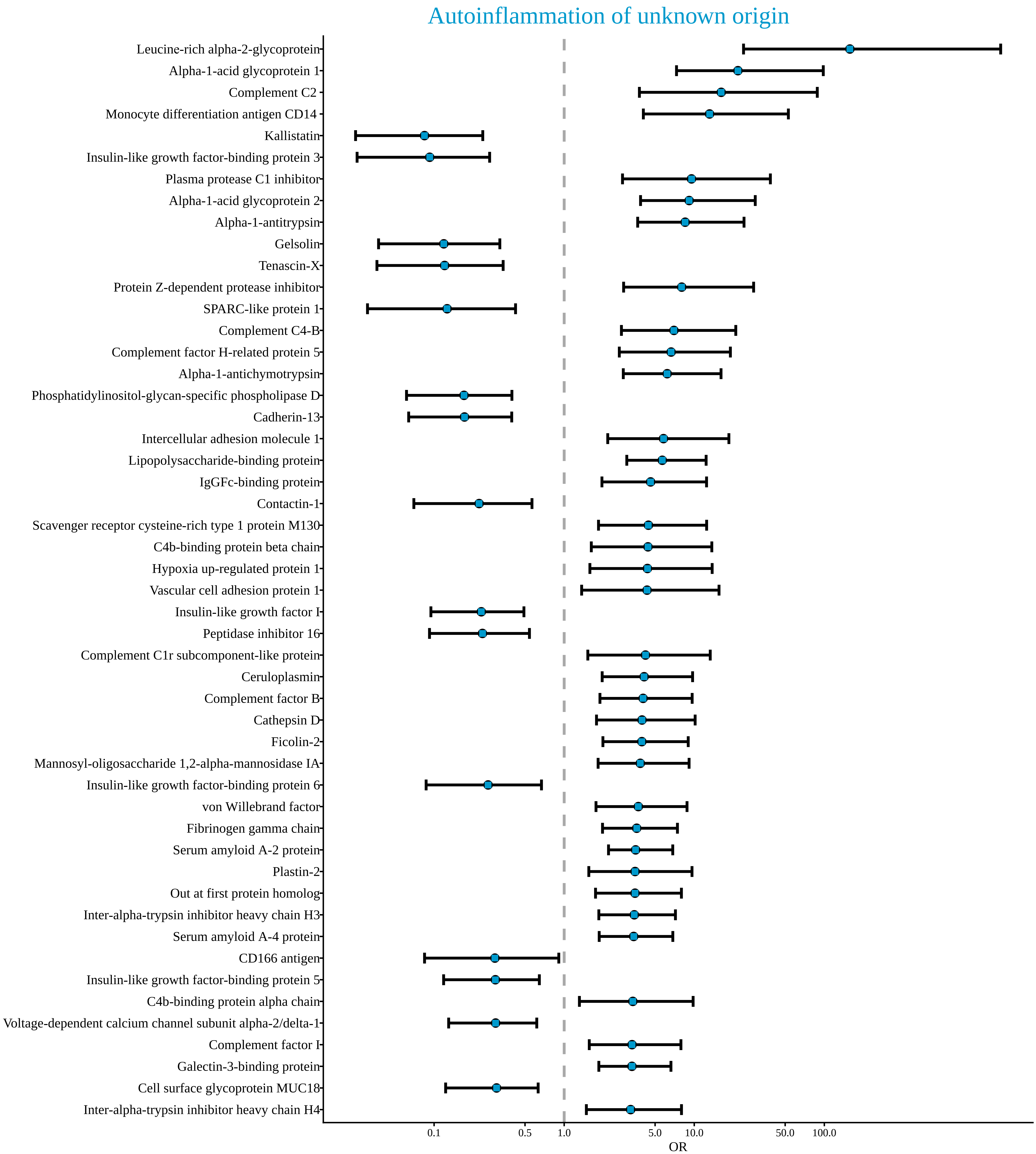
Autoinflammation of unknown origin
Leucine-rich alpha-2-glycoprotein
Alpha-1-acid glycoprotein 1
Complement C2
Monocyte differentiation antigen CD14
Kallistatin
Insulin-like growth factor-binding protein 3
Plasma protease C1 inhibitor
Alpha-1-acid glycoprotein 2
Alpha-1-antitrypsin
Gelsolin
Tenascin-X
Protein Z-dependent protease inhibitor
SPARC-like protein 1
Complement C4-B
Complement factor H-related protein 5
Alpha-1-antichymotrypsin
Phosphatidylinositol-glycan-specific phospholipase D
Cadherin-13
Intercellular adhesion molecule 1
Lipopolysaccharide-binding protein
IgGFc-binding protein
Contactin-1
Scavenger receptor cysteine-rich type 1 protein M130
C4b-binding protein beta chain
Hypoxia up-regulated protein 1
Vascular cell adhesion protein 1
Insulin-like growth factor I
Peptidase inhibitor 16
Complement C1r subcomponent-like protein
Ceruloplasmin
Complement factor B
Cathepsin D
Ficolin-2
Mannosyl-oligosaccharide 1,2-alpha-mannosidase IA
Insulin-like growth factor-binding protein 6
von Willebrand factor
Fibrinogen gamma chain
Serum amyloid A-2 protein
Plastin-2
Out at first protein homolog
Inter-alpha-trypsin inhibitor heavy chain H3
Serum amyloid A-4 protein
CD166 antigen
Insulin-like growth factor-binding protein 5
C4b-binding protein alpha chain
Voltage-dependent calcium channel subunit alpha-2/delta-1
Complement factor I
Galectin-3-binding protein
Cell surface glycoprotein MUC18
Inter-alpha-trypsin inhibitor heavy chain H4
0.1
0.5
1.0
5.0
10.0
50.0
100.0
OR

Supplementary Figure 6. Plasma proteomic parameters with high association with autoinflammation of unknown. Odds ratio and 95% confidence interval (point and error bars) of 50 highly-associated plasma proteomic parameters in patients with inflammation of unknown origin in relation to healthy individuals. Estimated by multivariable logistic regression adjusted by sex and age

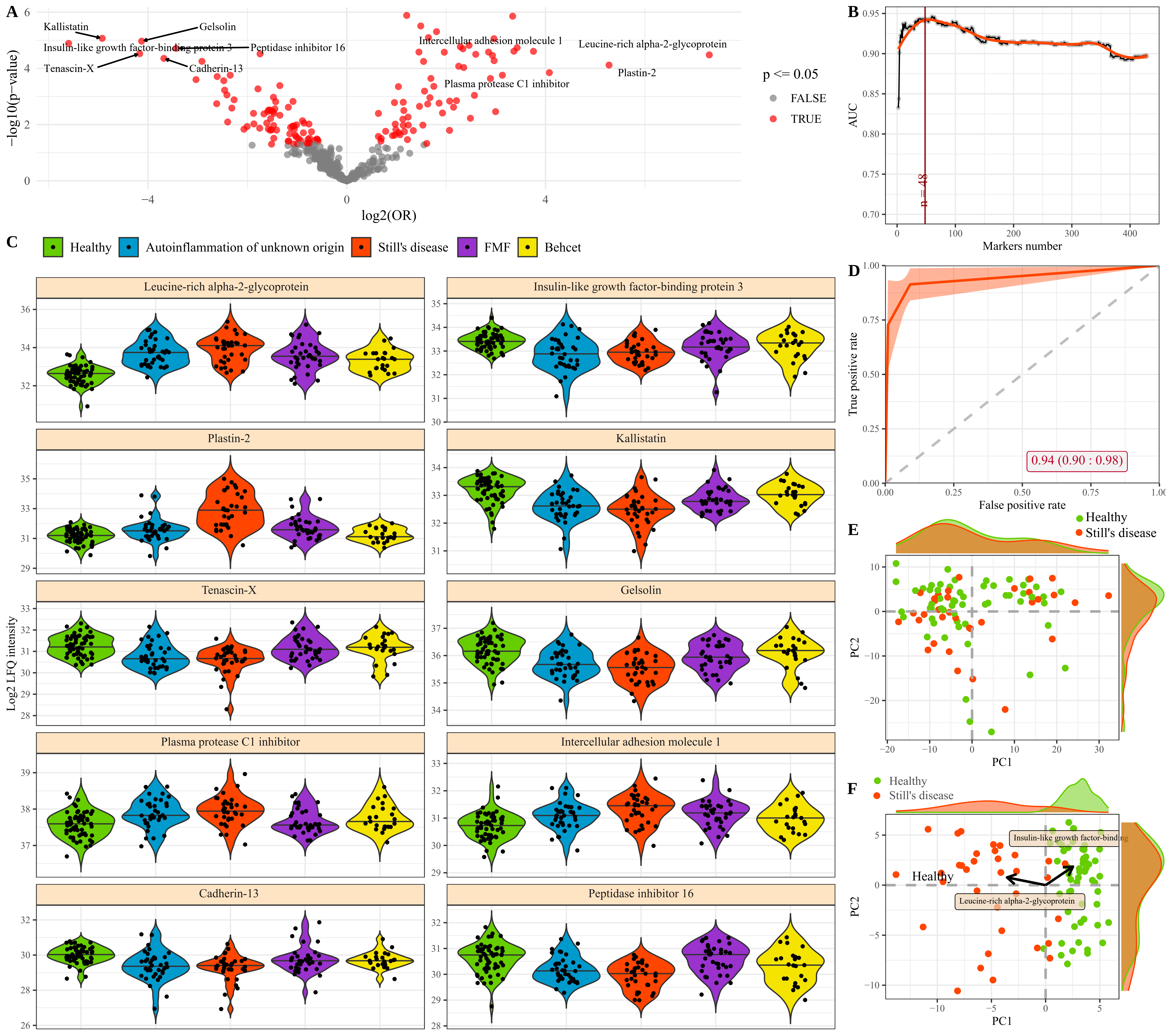

A
Kallistatin
Gelsolin
Insulin-like growth factor-binding protein 3
Peptidase inhibitor 16
Tenascin-X
Cadherin-13
Intercellular adhesion molecule 1
Leucine-rich alpha-2-glycoprotein
Plastin-2
Plasma protease C1 inhibitor
−log10(p−value)
log2(OR)
p <= 0.05
FALSE
TRUE
B
AUC
n = 48
Markers number
C
Healthy
Autoinflammation of unknown origin
Still's disease
FMF
Behcet
Leucine-rich alpha-2-glycoprotein
Insulin-like growth factor-binding protein 3
Plastin-2
Kallistatin
Tenascin-X
Gelsolin
Plasma protease C1 inhibitor
Intercellular adhesion molecule 1
Cadherin-13
Peptidase inhibitor 16
Log2 LFQ intensity
D
True positive rate
False positive rate
0.94 (0.90 : 0.98)
E
Healthy
Still's disease
PC2
PC1
F
Healthy
Still's disease
Insulin-like growth factor-binding
Leucine-rich alpha-2-glycoprotein
Healthy
PC2
PC1

Supplementary Figure 7. Plasma proteomic parameters associated with Still's disease. Patients with Still's disease (n=34) were compared against healthy individuals (n=58) by multivariate logistic regression of immunological parameters. Data from patients with autoinflammation of unknown origin (n=36), FMF (n=35) and Behçet (n=23) are shown as reference data only. **A)** Odds ratio and 95% confidence interval (point and error bars) of highly-associated plasma protein changes in patients with Still's disease in relation to healthy individuals. Estimated by multivariable logistic regression adjusted by sex and age. **B)** Average and 95% confidence interval (red line and red band) of 200 times 10 fold cross-validation to evaluate a sufficient number of plasma protein changes, based on ability to adequately discern between Still's disease and healthy individuals. **C)** Frequency for highly-associated plasma protein changes for Still's disease in relation to healthy individuals. Each dot represents a patient and each colour represents a condition. Bar indicates median, violin plot indicates data density. **D)** Average ROC curve with 95% confidence interval (red line and red band) of 10 fold cross-validation for Still's disease in relation to healthy individuals. ROC calculated using multi-variable logistic regression, adjusted by sex and age, considering the 53 plasma proteins with highest explanatory contribution. Area under ROC curve and confidence interval indicated on graph. **E)** First two PCA components of all plasma proteins in the dataset. Each dot represents an individual and each colour represents a condition. Histograms show distribution of values in Still's disease and healthy individuals. **F)** First two PCA components of 53 plasma proteins most highly associated for divergence between Still's disease and healthy individuals. Histograms show distribution of values in Still's disease and healthy individuals. The two arrows show the direction of distinct highly associated plasma proteins.

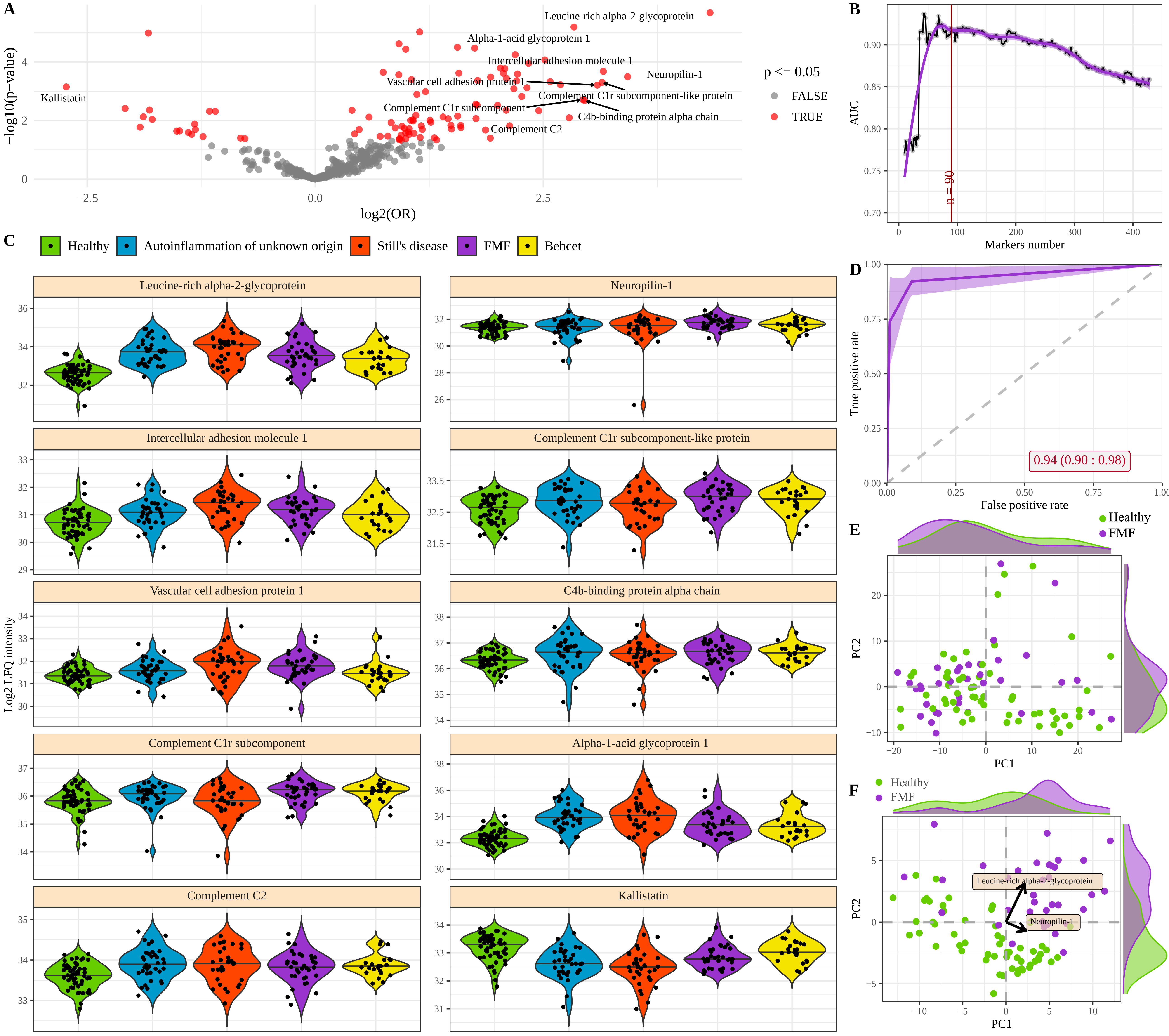

A
Leucine-rich alpha-2-glycoprotein
Alpha-1-acid glycoprotein 1
Intercellular adhesion molecule 1
Neuropilin-1
Vascular cell adhesion protein 1
Complement C1r subcomponent-like protein
Complement C1r subcomponent
C4b-binding protein alpha chain
Complement C2
Kallistatin
−log10(p−value)
log2(OR)
p <= 0.05
FALSE
TRUE
B
AUC
Markers number
n = 90
C
Healthy
Autoinflammation of unknown origin
Still's disease
FMF
Behcet
Log2 LFQ intensity
Leucine-rich alpha-2-glycoprotein
Neuropilin-1
Intercellular adhesion molecule 1
Complement C1r subcomponent-like protein
Vascular cell adhesion protein 1
C4b-binding protein alpha chain
Complement C1r subcomponent
Alpha-1-acid glycoprotein 1
Complement C2
Kallistatin
D
True positive rate
False positive rate
0.94 (0.90 : 0.98)
E
Healthy
FMF
PC1
PC2
F
Healthy
FMF
Leucine-rich alpha-2-glycoprotein
Neuropilin-1
PC1
PC2

Supplementary Figure 8. Plasma protein changes associated with FMF. Patients with FMF (n=35) were compared against healthy individuals (n=58) by multivariate logistic regression of plasma protein parameters. Data from patients with autoinflammation of unknown origin (n=36), Still's disease (n=34) and Behçet (n=23) are shown as reference data only. **A)** Odds ratio and 95% confidence interval (point and error bars) of highly-associated plasma protein abundance changes in patients with FMF in relation to healthy individuals. Estimated by multivariable logistic regression adjusted by sex and age. **B)** Average and 95% confidence interval (purple line and purple band) of 200 times 10 fold cross-validation to evaluate a sufficient number of best plasma proteins, based on ability to adequately discern between FMF and healthy individuals. **C)** Frequency for highly-associated plasma proteins for FMF in relation to healthy individuals. Each dot represents a patient and each colour represents a condition. Bar indicates median, violin plot indicates data density. **D)** Average ROC curve with 95% confidence interval (purple line and purple band) of 10 fold cross-validation for FMF in relation to healthy individuals. ROC calculated using multi-variable logistic regression, adjusted by sex and age, considering the 45 plasma proteins with highest explanatory contribution. Area under ROC curve and confidence interval indicated on graph. **E)** First two PCA components of all plasma proteins in the dataset. Each dot represents an individual and each colour represents a condition. Histograms show distribution of values in FMF and healthy individuals. **F)** First two PCA components of 45 plasma proteins most highly associated for divergence between FMF and healthy individuals. Histograms show distribution of values in FMF and healthy individuals. The two arrows show the direction of distinct highly associated plasma proteins.

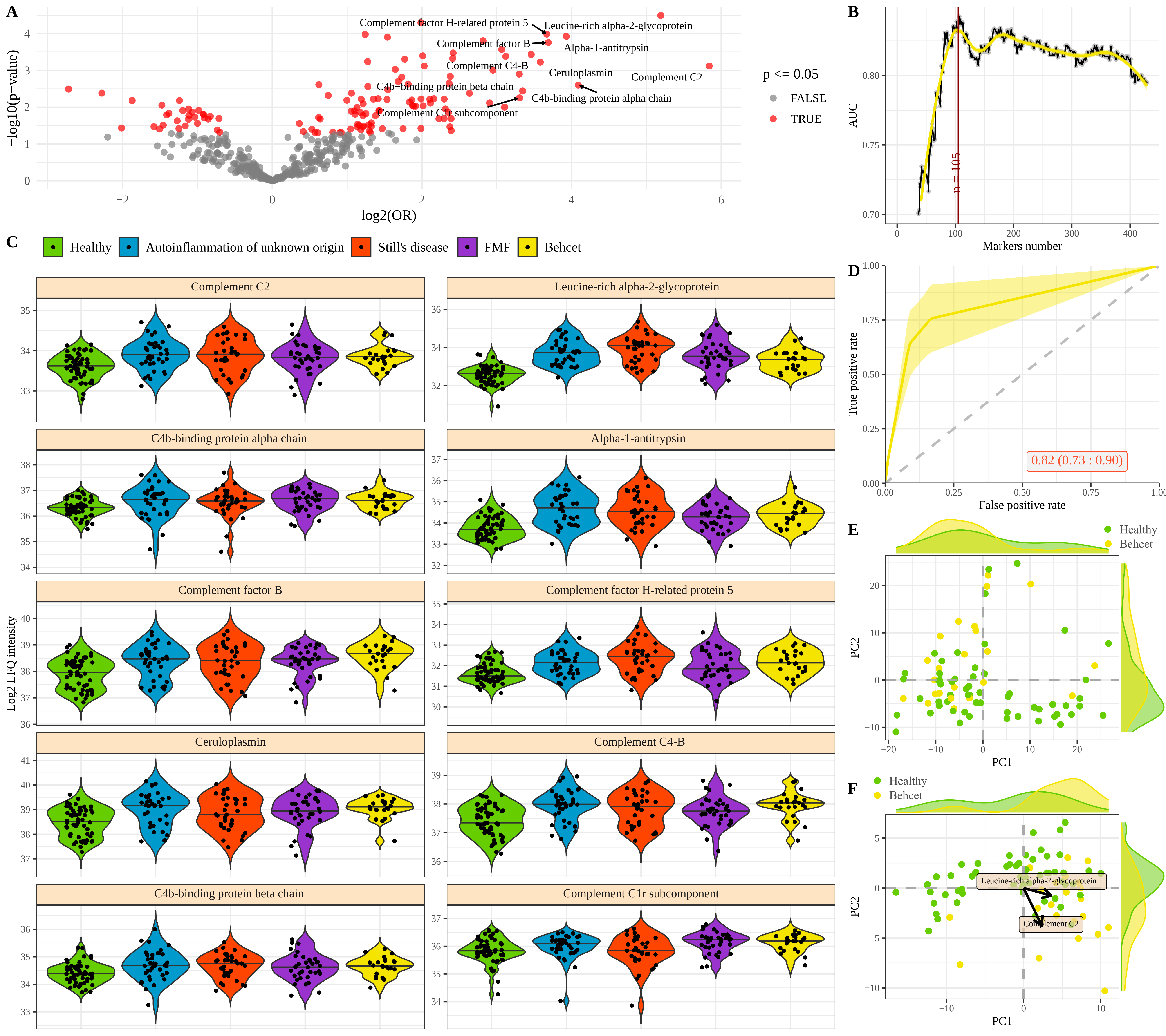

A
Complement factor H-related protein 5
Leucine-rich alpha-2-glycoprotein
Complement factor B
Alpha-1-antitrypsin
Complement C4-B
Ceruloplasmin
Complement C2
C4b-binding protein beta chain
C4b-binding protein alpha chain
Complement C1r subcomponent
−log10(p−value)
log2(OR)
p <= 0.05
FALSE
TRUE
B
AUC
n = 105
Markers number
C
Healthy
Autoinflammation of unknown origin
Still's disease
FMF
Behcet
Log2 LFQ intensity
D
True positive rate
False positive rate
0.82 (0.73 : 0.90)
E
Healthy
Behcet
PC1
PC2
F
Healthy
Behcet
Leucine-rich alpha-2-glycoprotein
Complement C2
PC1
PC2

Supplementary Figure 9. Plasma protein changes associated with Behçet's disease. Patients with Behçet's disease (n=23) were compared against healthy individuals (n=58) by multivariate logistic regression of plasma proteomic parameters. Data from patients with autoinflammation of unknown origin (n=36), Still's disease (n=34) and FMF (n=35) are shown as reference data only. **A)** Odds ratio and 95% confidence interval (point and error bars) of highly-associated plasma protein abundance changes in patients with Behçet in relation to healthy individuals. Estimated by multivariable logistic regression adjusted by sex and age. **B)** Average and 95% confidence interval (yellow line and yellow band) of 200 times 10 fold cross-validation to evaluate a sufficient number of best plasma proteins, based on ability to adequately discern between Behçet and healthy individuals. **C)** Frequency for highly-associated plasma proteins for Behçet in relation to healthy individuals. Each dot represents a patient and each colour represents a condition. Bar indicates median, violin plot indicates data density. **D)** Average ROC curve with 95% confidence (yellow line and yellow band) interval of 10 fold cross-validation for Behçet in relation to healthy individuals. ROC calculated using multi-variable logistic regression, adjusted by sex and age, considering the 112 plasma proteins with highest explanatory contribution. Area under ROC curve and confidence interval indicated on graph. **E)** First two PCA components of all plasma proteins in the dataset. Each dot represents an individual and each colour represents a condition. Histograms show distribution of values in Behçet and healthy individuals. **F)** First two PCA components of 112 plasma proteins most highly associated for divergence between Behçet and healthy individuals. Histograms show distribution of values in Behçet and healthy individuals. The two arrows show the direction of distinct highly associated plasma proteins.

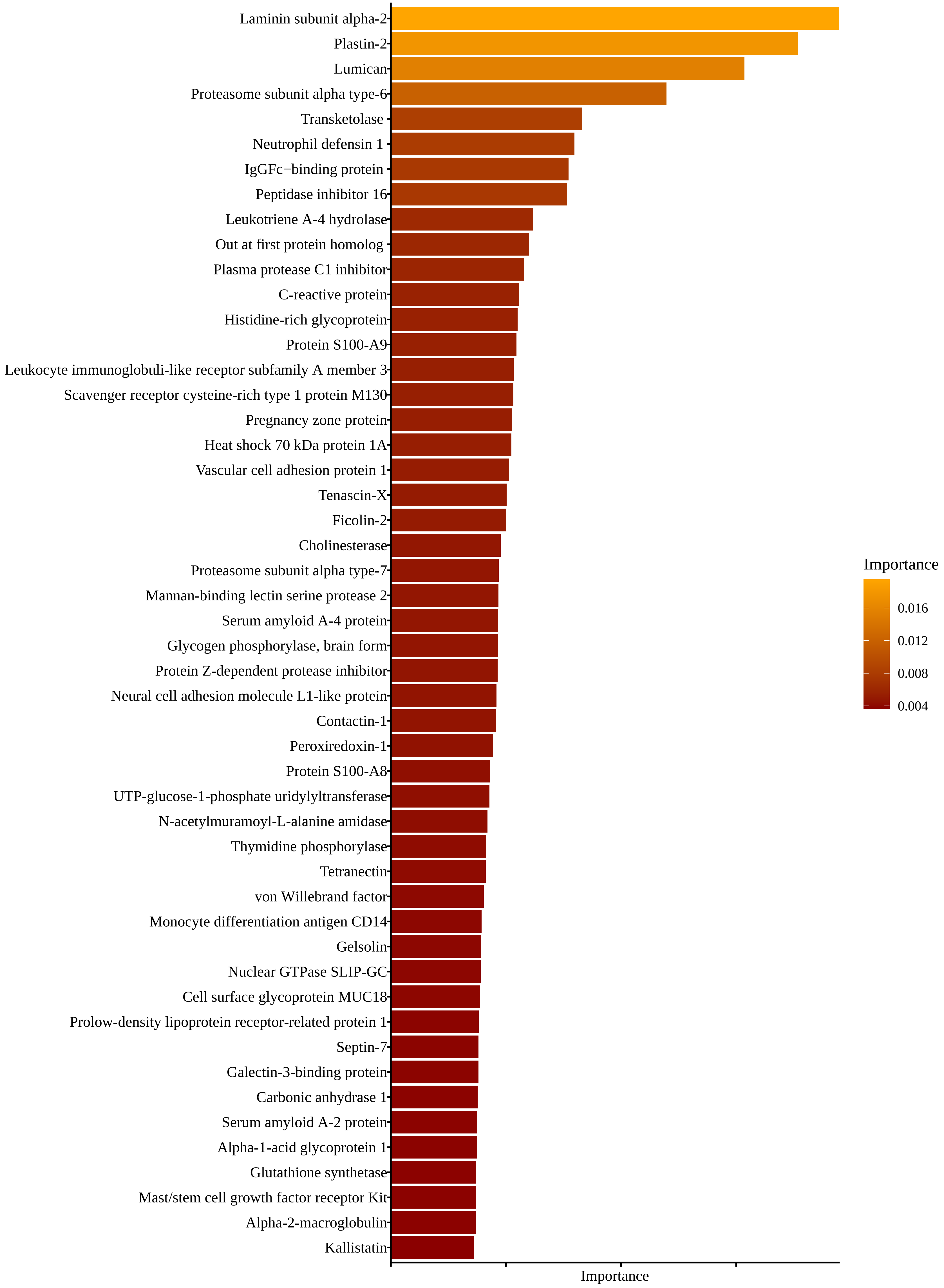

Laminin subunit alpha-2
Plastin-2
Lumican
Proteasome subunit alpha type-6
Transketolase
Neutrophil defensin 1
IgGFc−binding protein
Peptidase inhibitor 16
Leukotriene A-4 hydrolase
Out at first protein homolog
Plasma protease C1 inhibitor
C-reactive protein
Histidine-rich glycoprotein
Protein S100-A9
Leukocyte immunoglobuli-like receptor subfamily A member 3
Scavenger receptor cysteine-rich type 1 protein M130
Pregnancy zone protein
Heat shock 70 kDa protein 1A
Vascular cell adhesion protein 1
Tenascin-X
Ficolin-2
Cholinesterase
Proteasome subunit alpha type-7
Mannan-binding lectin serine protease 2
Serum amyloid A-4 protein
Glycogen phosphorylase, brain form
Protein Z-dependent protease inhibitor
Neural cell adhesion molecule L1-like protein
Contactin-1
Peroxiredoxin-1
Protein S100-A8
UTP-glucose-1-phosphate uridylyltransferase
N-acetylmuramoyl-L-alanine amidase
Thymidine phosphorylase
Tetranectin
von Willebrand factor
Monocyte differentiation antigen CD14
Gelsolin
Nuclear GTPase SLIP-GC
Cell surface glycoprotein MUC18
Prolow-density lipoprotein receptor-related protein 1
Septin-7
Galectin-3-binding protein
Carbonic anhydrase 1
Serum amyloid A-2 protein
Alpha-1-acid glycoprotein 1
Glutathione synthetase
Mast/stem cell growth factor receptor Kit
Alpha-2-macroglobulin
Kallistatin
Importance
Importance
0.016
0.012
0.008
0.004

Supplementary Figure 10. Key proteomic features driving machine learning-led disease identification. A multi-disease comparison was performed using a Random Forest algorithm to identify plasma proteomic characteristics with discriminating potential between patients with autoinflammation of unknown origin (n=36), Still's disease (n=34), FMF (n=35) and Behçet's (n=23). Healthy individuals were not used in the model generation, and are shown as reference data only. Model importance for the 50 highest associated plasma protein changes for disease discrimination.

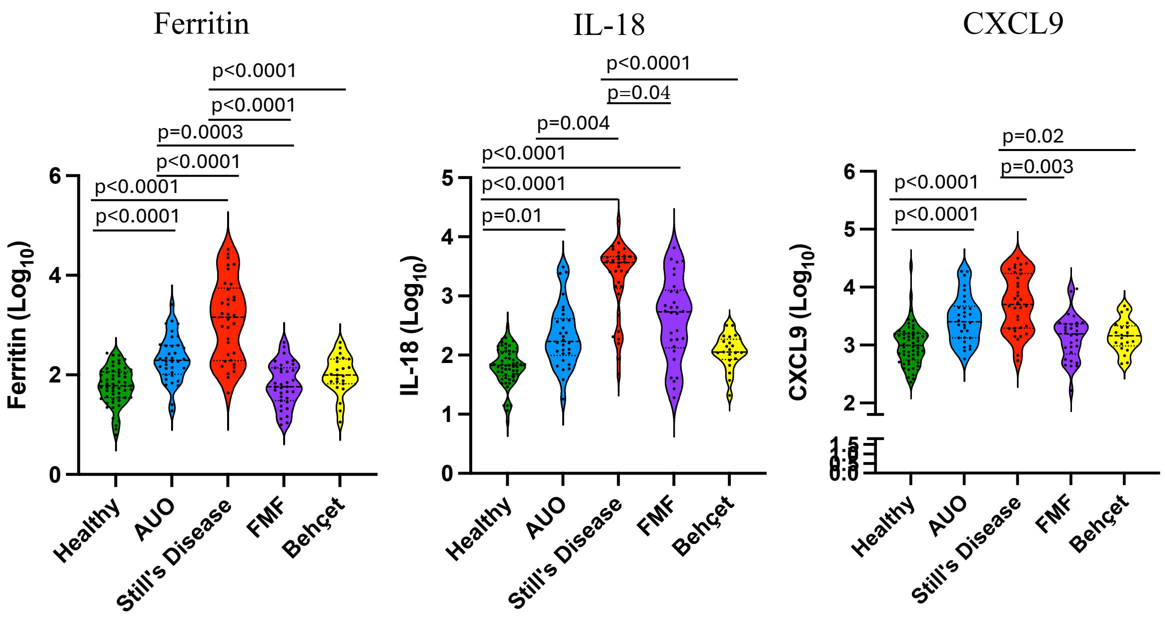

Ferritin
IL-18
CXCL9
p<0.0001
p<0.0001
p=0.0003
p<0.0001
p<0.0001
p<0.0001
p<0.0001
p=0.04
p=0.004
p<0.0001
p<0.0001
p=0.01
p=0.02
p=0.003
p<0.0001
p<0.0001
Ferritin (Log10)
IL-18 (Log10)
CXCL9 (Log10)
Healthy
AUO
Still's Disease
FMF
Behçet

Supplementary Figure 11. Quantification of Still's disease biomarkers. Quantification in plasma of ferritin (Chemiluminescent Microparticle ImmunoAssay-based assay; ng/mL), IL-18 (Luminex; pg/mL), and CXCL9 (Luminex; pg/mL), in healthy controls and in patients diagnosed with autoinflammation of unknown origin (AUO), Still's disease, familial Mediterranean fever (FMF) or Behçet's disease. Statistical analyses were performed after assessing the normality of the data distribution. Depending on the outcome, either a one way ANOVA followed by Tukey's multiple comparisons test or a Kruskal–Wallis test followed by Dunn's multiple comparisons test was applied.

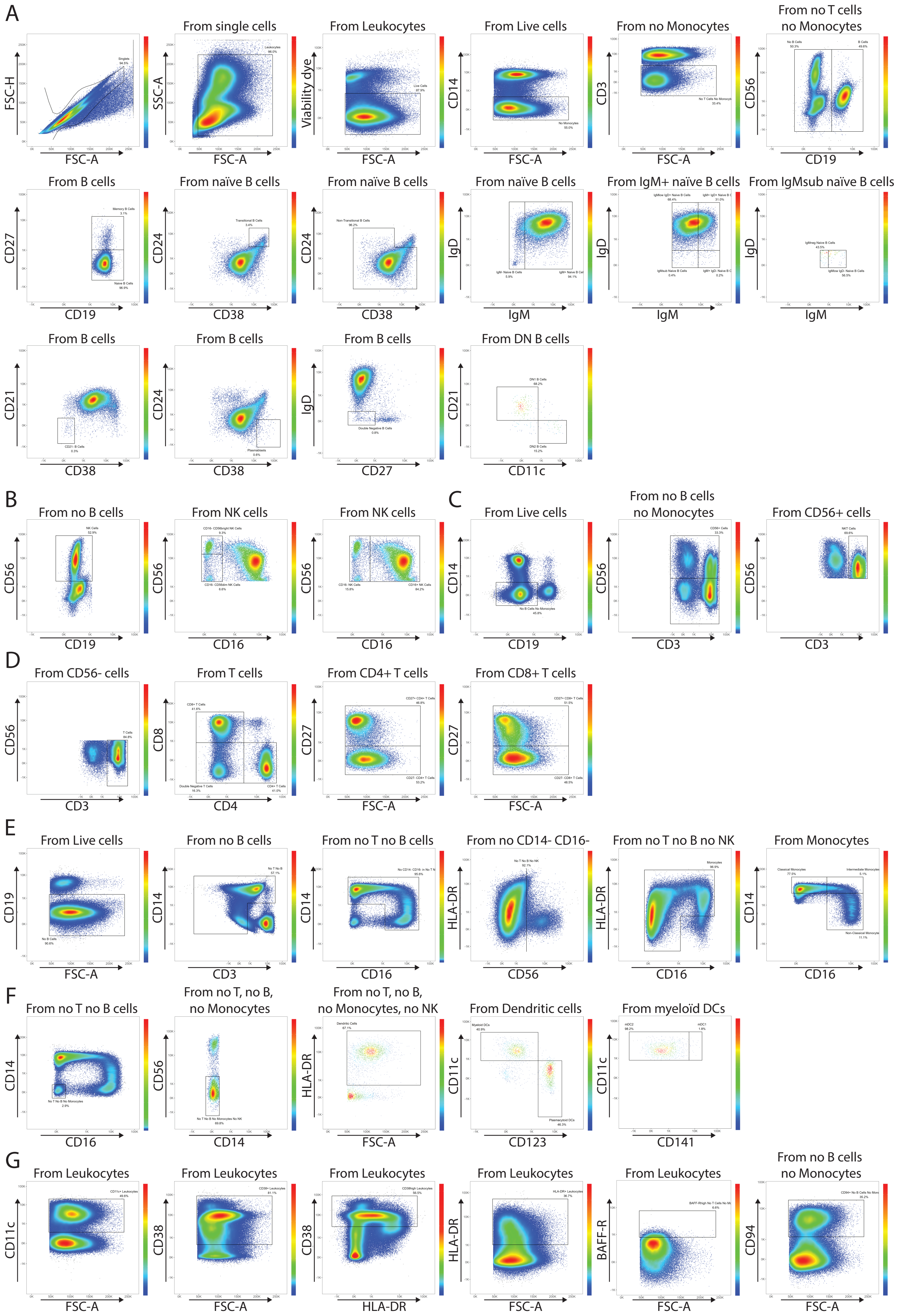
A
FSC-H
FSC-A
From single cells
SSC-A
FSC-A
From Leukocytes
Viability dye
FSC-A
From Live cells
CD14
FSC-A
From no Monocytes
CD3
FSC-A
From no T cells no Monocytes
CD56
CD19
From B cells
CD27
CD19
From naïve B cells
CD24
CD38
From naïve B cells
CD24
CD38
From naïve B cells
IgD
IgM
From IgM+ naïve B cells
IgD
IgM
From IgMsub naïve B cells
IgD
IgM
From B cells
CD21
CD38
From B cells
CD24
CD38
From B cells
IgD
CD27
From DN B cells
CD21
CD11c
B
From no B cells
CD56
CD19
From NK cells
CD56
CD16
From NK cells
CD56
CD16
C
From Live cells
CD14
CD19
From no B cells no Monocytes
CD56
CD3
From CD56+ cells
CD56
CD3
D
From CD56- cells
CD56
CD3
From T cells
CD8
CD4
From CD4+ T cells
CD27
FSC-A
From CD8+ T cells
CD27
FSC-A
E
From Live cells
CD19
FSC-A
From no B cells
CD14
CD3
From no T no B cells
CD14
CD16
From no CD14- CD16-
HLA-DR
CD56
From no T no B no NK
HLA-DR
CD16
From Monocytes
CD14
CD16
F
From no T no B cells
CD14
CD16
From no T, no B, no Monocytes
CD56
CD14
From no T, no B, no Monocytes, no NK
HLA-DR
FSC-A
From Dendritic cells
CD11c
CD123
From myeloïd DCs
CD11c
CD141
G
From Leukocytes
CD11c
FSC-A
From Leukocytes
CD38
FSC-A
From Leukocytes
CD38
HLA-DR
From Leukocytes
HLA-DR
FSC-A
From Leukocytes
BAFF-R
FSC-A
From no B cells no Monocytes
CD94
FSC-A

Supplementary Figure 12. Gating strategy applied to identify different cellular subsets and their activation markers. Gating strategy to identify **A)** B cells, **B)** NK cells, **C)** NKT cells, **D)** T cells, **E)** Monocytes, **F)** Dendritic cells, and their subsets. **G)** The gating strategy for all activation markers was calculated on leukocytes and applied to all cell subsets.

Supplementary Table 1. Power calculation for effect size

	Power	
OR	Without correction	Bonferroni correction
1.2	0.11	<0.01
1.5	0.38	0.01
1.8	0.66	0.07
2.0	0.80	0.15
2.5	0.96	0.44
3.0	0.99	0.72

Supplementary Table 2. Antibodies used in the study

Antibody	Clone	Company	Catalog number	Dilution
anti-human BAFF-R (BV650)	11C1	BD Biosciences	743572	60
anti-human CD10 (PE-Cy5)	HI10a	BioLegend	312206	40
anti-human CD11c (APC)	3.9	BioLegend	301614	25
anti-human CD123 (BB660-P)	7G3	BD Biosciences	customized	75
anti-human CD14 (PE-Cy5.5)	TuK4	eBioscience	MHCD1418	200
anti-human CD141 (BUV615-P)	1A4	BD Biosciences	customized	40
anti-human CD16 (BUV395)	3G8	BD Biosciences	563785	50
anti-human CD19 (BV510)	HIB19	BioLegend	302242	25
anti-human CD21 (BV786)	B-ly4	BD Biosciences	740969	50
anti-human CD24 (BV711)	ML5	BioLegend	311136	50
anti-human CD27 (BV750-P)	L128	BD Biosciences	customized	40
anti-human CD3 (PerCP-VIO 700)	REA613	Miltenyi Biotec	130-113-141	40
anti-human CD38 (PE/CF594)	HB-7	BioLegend	356630	100
anti-human CD4 (BUV496)	SK3	BD Biosciences	612936	50
anti-human CD40 (APC-R700)	5C3	BD Biosciences	565179	40
anti-human CD56 (BUV563)	NCAM16.2	BD Biosciences	612928	50
anti-human CD57 (FITC)	HNK-1	BioLegend	359604	100
anti-human CD80 (BB630)	L307.4	BD Biosciences	customized	40
anti-human CD86 (BUV737)	2331 (FUN-1)	BD Biosciences	612784	50
anti-human CD8a (BUV805)	RPA-T8	eBioscience	368-0088-42	75
anti-human CD94 (BV605)	HP-3D9	BD Biosciences	743950	50
anti-human HLA-DR (BV570)	L243	BioLegend	307638	40
anti-human IgD (PE-Cy7)	IA6-2	BioLegend	348210	75
anti-human IgM (BV421)	MHM-88	BioLegend	314516	100
Fixable Viability Dye eFluor780		eBioscience	65-0865-18	1000
