# Supplementary material for: Adult patients with autoinflammation of unknown origin partially phenocopy the immune presentation of Still’s disease
Source: Nat Commun. 2026 Apr 1;17:4670. doi: 10.1038/s41467-026-70895-1 (PMC13201634; doi:10.1038/s41467-026-70895-1)
Supplement: Supplementary file 3 — Description of Additional Supplementary Files [file 41467_2026_70895_MOESM3_ESM.pdf]

Description of Additional Supplementary Files

Supplementary Data 1. Clinical resource for SAID patients. Clinical and demographic features for each included SAID patient in the cohort.

Supplementary Data 2. Gated populations with gating hierarchy. Gating hierarchy and marker definitions for the 208 immune variables (frequency of population within parent or super-parent) used in the study.

Supplementary Data 3. Flow cytometry data resource for SAID patients. Raw flow cytometry cell frequency for immunological subsets across the patients and controls used in this study, together with demographic features.

Supplementary Data 4. Mass spectrometry data resource for SAID patients. Raw protein abundance across the patients and controls used in this study, together with demographic features.
